# How Does the pH of Tree Bark Change with the Presence of the Epiphytic Bryophytes from the Family Orthotrichaceae in the Interaction with Trunk Inclination?

**DOI:** 10.3390/plants11010063

**Published:** 2021-12-25

**Authors:** Markéta Kovářová, Petr Pyszko, Vítězslav Plášek

**Affiliations:** 1 Department of Biology and Ecology, Faculty of Science, University of Ostrava, Chittussiho 10, 71000 Ostrava, Czech Republic; kovarova.marketa@gmail.com (M.K.); petr.pyszko@osu.cz (P.P.); 2 Institute of Biology, University of Opole, 45-040 Opole, Poland

**Keywords:** acidification, dust deposition, inclination, Orthotrichaceae, phorophytes

## Abstract

The pH of tree bark is affected by many factors, amongst them epiphytic bryophytes changing in their active state environment. Thus, we hypothesized that bryophytes can change bark acidity, dependently of the inclination of the branches, as inclination affect the water regime and particle deposition. We measured the pH under bryophyte cushions and compared it to nearby naked bark. Additionally, we compared results with experimental bark covering with neutral cover. We found that the pH of naked bark declines with decreasing inclination of trunks. Although bryophyte cover did not generally change the pH of the bark, there was a significant interaction with inclination: with higher inclination, bryophytes decrease the pH reaction of bark, while with lower inclination they increase it. One possible explanation may lie in changes to alkaline particle deposition, or conversely in the acidification of the bark by leaching. In addition, an experiment with a neutral cover showed that naked bark covering would substantially increase pH. As, on average, bryophytes do not change the pH of bark, there can be mutual interference between the alkalizing effect of the bark cover itself and the acidifying biological effect of bryophytes.

## 1. Introduction

Tree bark varies in pH [1]. Bark pH is influenced by several biotic and abiotic factors and is species-specific among woody plants [2,3,4]. There may not be a direct relationship between pH and tree species [5]. However, tree species can affect pH, increase (beech) or decrease it (spruce) [6]. Tree species also determines the shape of the leaves, the angle of connection of the branches, the architecture of the bark, and thus the amount and type of substances leached from the leaf area and the bark [7,8]. Another condition that affects pH reaction is the size of the tree (the crown area, the total number of branches), which is closely related to its age and circumference [9,10]. The age of the tree affects the texture of the bark [11] which affects the drying time of bark moisture [12,13] often species-specifically [14,15]. Abiotic factors influencing the pH of the bark are the type of precipitation, seasonality [16,17], the amount and intensity of precipitation [18], the deposition of alkaline dust particles on the tree surface, their aqueous solutions [1,19], and wind [20].

The presence of epiphytic bryophytes on the trunk surface of tree phorophytes should also affect the pH of the bark. Bryophytes are typical poikilohydric organisms [21] absorbing relatively acidic rainwater [22] and thus partially protecting the bark below from its acidifying influence. Epiphytic bryophytes also affect the composition of the flush by removing nitrogenous substances from the leachate [23]. Dust particles can be deposited on the surface of bryophytes, similarly to bark [24], and these can cause higher alkalinity of the flush by subsequent leaching. Epiphytic bryophytes often grow in the grooves of bark [25] and their presence can change the its direction of water flow and water capacity [26], thus affecting which parts of the bark will be washed by water or affected by the leaching of substances from the bryophyte. Due to the longer retention of water by bryophytes, the bark under them is softer and disintegrates more easily [27]. Deeper layers with a different composition thus reach the surface of the bark. Deeper bark layers contain living secondary wood, phelloderm, dead wood, and the part of the bark where substances flow from the roots. Therefore it has a different chemical character, e.g., acidity, metals contained, etc. [2]. 

The relationship between pH and the occurrence of epiphytic bryophytes has already been studied from various perspectives. Spore germination and gametophyte growth were studied concerning the impact of pH reaction [28,29,30]. Wiklund and Rydin found that in conifers with lower bark pH, spore germination takes longer or is completely inhibited [31]. Epiphytic bryophytes prefer trees with rather a neutral pH [32,33]. They grow most often on deciduous trees such as poplars and willows, which have bark with a relatively higher, subneutral pH [24,34,35,36]. Older trees with a higher bark pH are more often overgrown by epiphytes [37,38,39]. 

All those studies follow up the effect of bark pH on bryophytes. The possible reverse effect of epiphytic bryophytes on bark pH has only rarely been studied. Some studies measure only the pH of the bark without bryophytes and suggest that the pH of bark at a site of bryophyte growth may differ from that of bark without bryophytes [40]. Other studies found a significant increase of pH under bryophytes in comparison to bark without bryophytes. However, using mixed samples of bark, one study pointed out the necessity of paired samples in future work [41].

We aimed to study pH changes under bryophytes by comparing paired samples of bark under bryophyte cover and naked bark. Specifically, we are interested in the role of trunk inclination on the change of pH under bryophytes, as the impact of the flush of leachate or dust particles deposition may vary with changing inclination, and both are strongly affected by epiphytic bryophytes as mentioned above. In addition, we compare the results with a neutral cover of the bark itself without bryophytes. We chose the family Orthotrichaceae as a model group of organisms with regard to their frequency in the studied area and their mostly epiphytical growth [42].

Specifically, we aimed to find out how:

(i) Inclination influences the pH of the naked bark of selected tree species.
(ii) The pH of naked bark differs from the pH of the bark covered by bryophytes and inclination affects the pH difference within paired samples.
(iii) The pH of bark changes after with neutral cover in comparison to with bryophyte cover.

## 2. Results

Altogether, we obtained 115 paired samples of bryophytes and nearby naked bark from three studied phorophyte species: *Acer pseudoplatanus* L. (*n* = 65), *Fraxinus excelsior* L. (*n* = 15), *Salix caprea* L. (*n* = 35). We identified nine species of Orthotrichaceae: *Lewinskya affinis* var. *affinis* F. Lara, (Brid.) Garilleti and Goffinet (*n* = 44), *Lewinskya speciosa* (Nees) F. Lara, Garilleti and Goffinet (*n* = 25), *Nyholmiella obtusifolia* (Brid.) Holmen and E. Warncke (*n* = 10), *Orthotrichum pallens* Brid. (*n* = 11), *Orthotrichum pumilum* Sw. (*n* = 8), *Ulota bruchii* Hornsch. ex Brid. (*n* = 11), and *Ulota crispa* (Hedw.) Brid. (*n* = 6). Another nine samples were excluded from the analysis because they belonged to species of bryophytes with fewer than 6 samples (*Orthotrichum patens* Bruch ex Brid., *n* = 4; *Orthotrichum striatum* Hedw., *n* = 2) or were collected from another phorophyte (*Sorbus aucuparia* L., *n* = 3).

The pH measured by the in situ method ($\bar{x}$ = 4.79 ± 0.481) was significantly lower (V = 325, *p* < 0.001) than after grinding ($\bar{x}$ = 5.54 ± 0.475), but they strongly correlated (r = 0.999, t = 120.90, *p* < 0.001).

**Figure 1:**
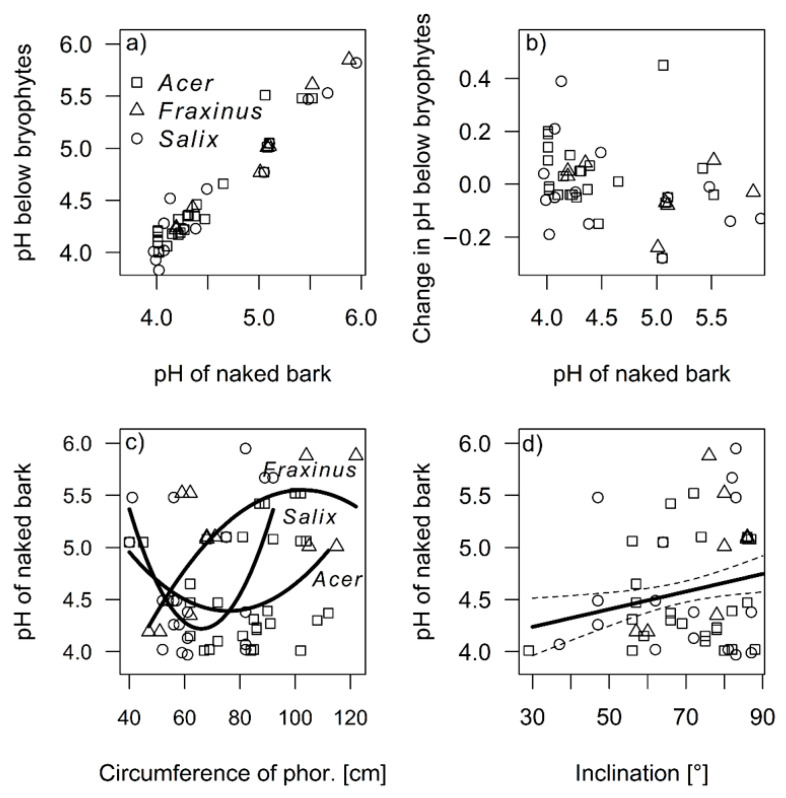
Correlation between pH near bryophytes and below bryophytes (**a**). Change in pH below bryophytes was correlated with the pH in the control samples near bryophytes (**b**). The pH near bryophytes changes with the circumference of phorophytes (**c**), and inclination (**d**). The dashed lines represents the 95% confidence intervals (CI). The trend curves were created based on generalized linear models with Gamma distribution.

The pH in the control samples of naked bark ($\bar{x}$ = 4.57 ± 0.651) and below bryophytes ($\bar{x}$ = 4.57 ± 0.550) did not differ (V = 3679.50, *p* = 0.337), and strongly correlated (τ = 0.814, z = 12.53, *p* < 0.001) (Figure 1a). However, the change in pH below bryophyte was negatively correlated with the pH in the control samples (τ = −0.157, z = −2.39, *p* = 0.017), i.e., with decreasing pH of naked bark, the pH under bryophytes increased, whereas with increasing pH of naked bark, the pH under bryophytes decreased (Figure 1b). 

The pH of naked bark changed with the circumference of the phorophyte (df = 105, F = 6.66, *p* = 0.002), nearly significantly with the species of phorophyte (df = 105, F = 2.38, *p* = 0.097), and with the interaction between both variables (df = 105, F = 3.32, *p* = 0.013). With increasing circumference, the pH had unimodal trend for *Acer* sp. and *Salix* sp. The lowest pH was on the bark of middle-sized phorophytes, but was an increasing trend for *Fraxinus* sp. (Figure 1c). Furthermore, the pH increased with inclination (df = 105, F = 7.94, *p* = 0.006) (Figure 1d). 

**Figure 2:**
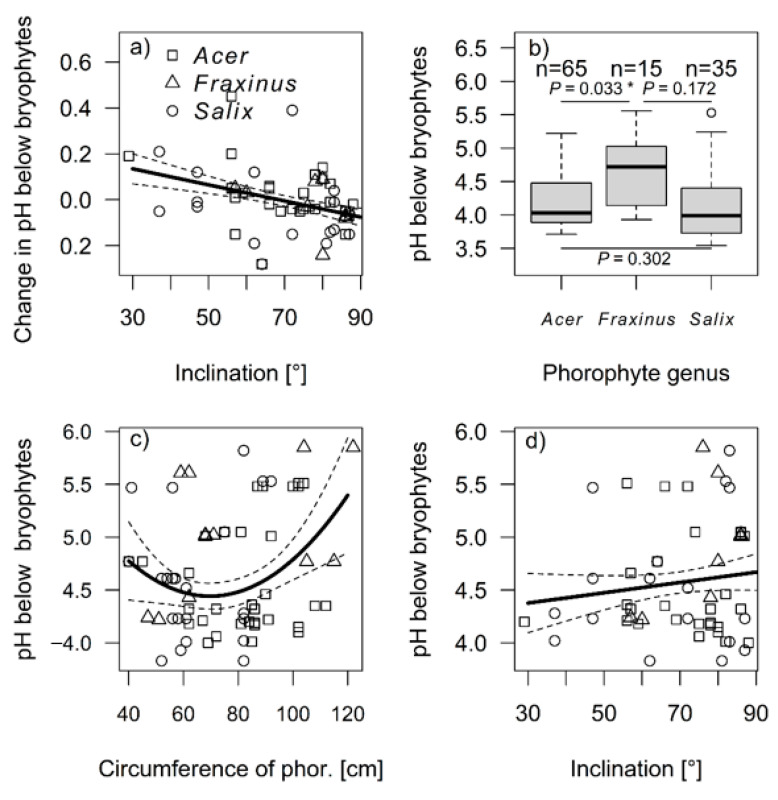
The difference in pH below bryophytes (in comparison to control samples near bryophytes) along the gradient of the inclination (**a**). The pH below bryophytes compared amongst the species of phorophytes (**b**). The pH below bryophytes along the gradient of the circumference of phorophytes with *p*-values for pairwise comparisons (**c**), and along the gradient of the inclination (**d**). In the scatter plots, the dashed lines represent the 95% CI. The trend curves were created based on generalized linear models with Gamma distribution. The asterisk (*) indicates that the value is significant.

The change in pH below bryophytes decreased strongly with increasing inclination (df = 113, F = 20.14, *p* < 0.001). With low inclination, the pH under bryophytes increased in comparison to control samples of naked bark, whereas with increasing inclination, the pH under bryophytes decreased in comparison with the control samples (Figure 2a). As a result, the pH below bryophytes differed among phorophyte species (df = 109, F = 3.22, *p* = 0.044) (Figure 2b), and unimodally changed with the circumference of phorophytes (df = 109, F = 5.74, *p* = 0.004) (Figure 2c), but did not change with inclination (df = 109, F = 0.89, *p* = 0.324) (Figure 2d). 

**Figure 3:**
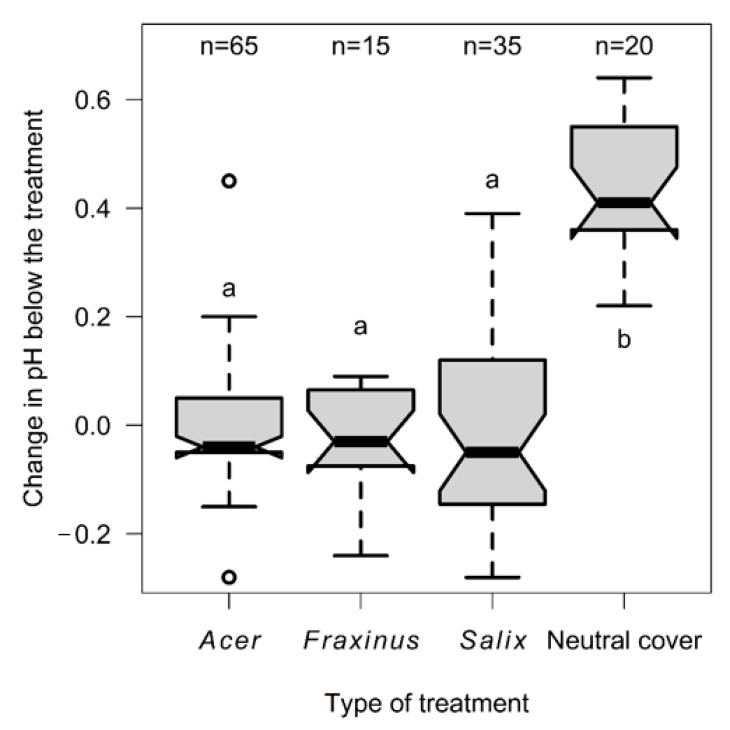
The difference in the change in pH (in comparison to control samples) below bryophytes and the experimental neutral cover with different labels for significantly different groups (α = 0.05; *p*-values for pairwise comparisons of neutral cover with other groups were always *p* < 0.001). Different letters a, b indicate groups with significantly different pH in pairwise comparison. ° rep-resents outliers.

In the experiment with neutral cover, the tree bark after the first control had significantly higher pH (df = 20, t = −17.50, *p* < 0.001) than before experiment: before experiment ($\bar{x}$ = 4.56 ± 0.453), after first control ($\bar{x}$ = 4.99 ± 0.423). This difference was explainable neither by circumference (df = 18, F = 0.59, *p* = 0.451), nor by inclination (df = 18, F = 0.08, *p* = 0.783). The differences between first and second control (V = 76.5, *p* = 0.181) and between second and third control (df = 20, t = −0.37, *p* = 0.712) were not significant. Furthermore, the Wilcoxon test showed that the change to pH in the experiment did not correspond to the differences between the pH of bark under bryophytes and the pH of naked bark (W = 41, *p* < 0.001) (Figure 3). 

## 3. Discussion

### 3.1. Drawbacks of Study

The research aimed to compare the pH of tree bark covered with bryophytes with naked bark to determine whether bryophytes affect the pH of the bark. The major weak point is the possibility that the bryophytes may select spots with more suitable conditions for growth within the tree [40], and thus spots with bark pH deviating from the surrounding pH. To reduce this bias, we measured the pH of paired samples of naked bark from as close as possible to the sampled bryophyte, and also considered the similarity of other conditions (e.g., texture). On the other hand, the paired sampling ruled out the measurement of trees with the absence of bryophytes. 

Another drawback was that the pH measurement was performed in situ. This method has the advantage that the measured pH is not affected by deeper layers of bark, contrarily to the method of the drying and grinding of a bark sample which is dissolved before the pH is determined [2,43]. The disadvantage to this method was the outflow of solvent from the surface and thus the impossibility of more accurate standardization of its quantity [40]. We thus compared both methods: the in situ method provided lower results (pH was lower for 0.75 in all samples), but both methods strongly correlated, so we assumed that both methods are commutable. Furthermore, good comparability of this method with the results of other methods was previously reported [43,44]. 

### 3.2. Effect of Inclination on pH of the Bark

The essential point of our research was to find out if inclination affects the pH of the naked bark of selected tree species. The pH decreases with decreasing inclination, similar to the results found by other researchers [7,24]. Precipitations leach and wash away from the surface of the trees’ settled cations (e.g., K^+^, Mg^2+^, Ca^2+^, P^+^, Mn^2+^), increasing their content in the flowing water [17,19,45], and consequently increasing the pH of the water. Significant leaching occurs on less inclined surfaces (usually branches). The water enriched with alkaline particles from the flush or leachate [18,19] then flows down through the surfaces with a higher inclination (along the trunk), increasing their pH [24,46]. As a result, precipitation decreases pH in higher parts with lower inclination, and increases pH in the lower parts of the tree with higher inclination [18,24]; to the same was true in our data. This acidification can be promoted by the run-off of relatively acidic rainwater during heavy rains [18].

Bryophytes on trees absorb quite a large amount of water [26,47] and can decrease the velocity of flowing water. Slowing down (e.g., due to low temperature) leads usually to accumulation of water on plant parts with low inclination [17,48]. However, bryophytes should also slow down the flow of water on plant parts with a higher inclination, where usually the fast flow of water is insufficient for leaching. On the other hand, on plant parts with lower inclination, longer retention of water related to longer leaching could be outweighed by the retention of cations by the bryophytes themselves and also by the higher deposition of dust particles on surfaces with bryophytes in lower inclinations [7,8]. This hypothesis was supported by results, as the bryophytes appeared to acidify the pH at high inclinations with a high pH and alkalinize it at low inclinations with a low pH. 

An experiment with neutral bark cover showed that the covering itself leads to higher pH in all samples, whereas the pH of the bark at the bryophyte was not different from the pH of the naked bark, contrarily to the previous researches [41]. We hypothesize that the increase of pH under neutral cover may have been caused by prolonged retention of water [49] and thus the increased potential for the leaching and retention of alkaline substances caused by the cover [15]. We thus assume that if the pH under the bryophytes changed only due to the covering of the bark, it should be significantly higher than it is in locations without such coverings. Thus, we can conclude that the mechanical effect of bark covering by bryophytes was mitigated by the assumed acidifying biological effect of bryophytes. If there are epiphytes on the surface of the bark, they should assimilate N (NHx, NOx) from the leachate [50], resulting in nitrogen and consequently also a pH decrease in the flush [23,51]. Contrarily, higher pH under bryophytes of up to 0.5 was recorded [41], but this result was not compared with the pH of naked bark. Such a comparison can disclose that the pH is the result of the above-mentioned contradictory effects. 

The seemingly small impact of bryophytes on pH can be the result of two strong opposing forces (the alkalinizing effect of cover, and the acidifying effect of bryophytes). Thus, deciphering the effects of bryophytes on bark pH in further research may be of importance, as the pH of the bark can have a significant impact on the growth of other epiphytic organisms, such as lichens [1], epiphytic ferns [52], or orchids [53], and pH of the stemflow has broad consequences for the biota near the trees, including epigeic and endogenous species [54], as well as the composition of microbiota [55]. The content of biologically active substances in bryophytes depends on the ecological conditions of growth. The pH of the bark can affect also the amount and type of biologically active substances, which may strongly vary in the bryophytes [28,56,57,58], and vice versa.

The circumference of the phorophyte as a proxy for its age is also related to changes in pH. Old phorophytes over 70 cm have a bark pH up to subneutral, which suits bryophytes [59]. Their preference for older trunks [13,40,60] is caused by the changes in the volume and composition of the flowing water, as bryophytes often occur in the grooves and crevices of the bark, where large quantities of water flow [13]. The greater thickness of phorophyte bark [12] and its more pronounced texture [11,13] result in longer wetting [15], increasing the amount of leached cations with the increasing size of the tree [49,61]. Thus, the observed change in pH in connection with the diameter may be caused by a species-specific change in bark architecture during tree growth [11]. With increasing circumference, the pH of the bark increased in *F. excelsior* as previously reported [34]. Bark pH of *A. pseudoplatanus* and *S. caprea* decreased, but from circumference of 60–70 cm, it increased [59]. 

## 4. Materials and Methods

### 4.1. Study Design

The study area (Lysá hora Mt.) was located in the center of Moravian-Silesian Beskydy Mts, Czech Republic (49.546N, 18.447E) [62]. In the years of study (2019–2020) we noted precipitation of 1467, 1897 mm, respectively. The pH of the rainfall was 5.28 and 5.28, respectively. The composition of cations was: Na^+^ (138, 124 ug/L), K^+^ (54, 76 ug/L), NH_4_^+^ (743, 673 ug/L), Mg^2+^ (34, 36 ug/L) and Ca^2+^ (203, 205 ug/L). The annual composition of anions in rainfall was: Cl^−^ (262, 329 ug/L), NO^3−^ (1686, 1246 ug/L) and SO_4_^2−^ (1217, 1140 ug/L) [63,64].

The pedological structure of the area consisted of mountain podzols and cambisol podzols (acid soils pH = 5–5.9). In the floodplains of streams there are gley fluvial soils on non-calcareous sediments (pH = 5–5.9) [65]. In occasionally exposed formations of sandstones, remnants of the Baden surface can be found [66]. The main type of vegetation in the study area was mixed forest. The crucial coniferous tree species were spruce *Picea abies* accompanied by deciduous trees, mainly *Acer pseudoplatanus*, *Fraxinus excelsior*, and *Salix caprea*, supplied by *Tilia cordata*, and *Sorbus aucuparia*. 

The research was conducted in the summers of 2019 and 2020. Bryophytes from the family Orthotrichaceae were chosen as a model group of organisms, with regard to their frequency in the studied area and because they grow mostly epiphytically [42,67]. All deciduous trees were searched for bryophytes. The main phorophytes were *A. pseudoplatanus*, *F. excelsior*, and *S. caprea*. Only a few samples of bryophytes were found on other tree species. Bryophytes were sampled from phorophytes at breast height (about 140 cm above the ground). To make the samples of the bark under the bryophytes comparable with naked bark in terms of pH, we selected paired samples of naked bark from the conditions as similar as possible, i.e., from the same height, bark texture, tree diameter at breast height (DBH) [2], inclination, and exposition. The pH of samples of the bark under bryophytes and the paired naked bark samples were measured by the in situ method, according to [40]. The bark was firstly dampened by 1 M potassium chloride solution (KCl). Then, the pH of the surface was measured by an ExStik™ flat surface electrode pH meter [1]. We allowed enough time (10 s) for the pH meter reading to stabilize [40,44]. The pH meter was calibrated in the prescribed solutions of pH 4 and pH 7 with a frequency of 15 measurements, as determined by the manufacturer. For the validation of our method, we took 25 paired samples to a laboratory for a standard method based on grinding and dissolving to measure pH [2,44]. Furthermore, we evaluated the bark texture recognizing three levels: smooth bark without grooves (*n* = 27), relatively smooth bark with some grooves (*n* = 42), and rough bark with plenty of grooves (*n* = 46) [34]. The degree of shading was also estimated on a three-level gradient: exposed (*n* = 6), semi-shaded (*n* = 66), and shaded (43). Using meters, we measured the exact height of samples above ground in cm ($\bar{x}$ = 142.74 ± SD = 4.202), DBH to approximate the age of the tree in cm ($\bar{x}$ = 24.21 ± SD = 5.509), and the area of bryophyte cover within the tree in cm^2^ ($\bar{x}$ = 192.78 ± SD = 83.156). Inclination was measured by a Pieps 30° plus XT digital inclinometer in degrees (29–88°, $\bar{x}$ = 69.56 ± SD = 14.882). The exposition was measured by a magnetic compass. Bryophytes collected in the field were determined in the laboratory using a binocular magnifier and a microscope. Their nomenclature was arranged according to [42,67].

The experiment with neutral cover took place during 2020. In February, we measured the pH at DBH of twenty haphazardly selected phorophytes. Then the bark was covered by a hydrophilic elastic bandage used as a cover and made of water-absorbing material similar to bryophytes, thus simulating their presence. After three months, the cover was removed, the pH was remeasured at the same spot, and the bark was covered again. Two more controls took place after three months, i.e., in August and in November, when the cover was removed.

### 4.2. Statistical Analysis

We processed the data in R 4.0.2. Firstly, we compared in situ method of pH measurement with a method based on the grinding of dried bark samples. We used a paired Wilcoxon signed-rank test to compare if either method gives a higher level of pH and use the Pearson parametric correlation test to investigate whether both methods’ results correspond with each other.

We compared the pH in the control samples near bryophytes and the pH below the bryophytes by a paired Wilcoxon signed-rank test, and we tested the correlation between them by a Kendall non-parametric correlation test. Then, we used the same correlation test to compare the pH in the control samples near the bryophytes with the difference between these samples and the pH below the bryophytes to determine the change in pH below bryophytes.

Then, we analyzed which explanatory variables affected the pH in the control samples near bryophytes by a generalized linear model with Gamma distribution. We used as explanatory variables: phorophyte species, bryophyte species, the texture of bark, DBH, bryophyte cover, and inclination. We used also the polynomes of the second degree of each continuous explanatory variable and their potential interactions with phorophyte or bryophyte species. The final model was constructed by stepwise selection based on the Akaike information criterion (AIC). Then, we used the same approach (using the same explanatory variables) to build the models for change in pH below bryophytes and for the pH below the bryophytes. For the final models, we checked the possible collinearity of variables by a variance inflation factor, potentially excluding those exceeding the threshold > 2.

We compared our results to the experiment with a neutral cover. By a series of paired *t*-tests or Wilcoxon signed-rank tests (selected according to the Shapiro–Wilk normality test) we first compared the pH levels of the bark before experimental treatment and during the first control, then between each control. Furthermore, we tried to explain the change in pH by a linear model, using the explanatory variable significant in the previous steps (i.e., inclination and circumference of the phorophytes). Then we compared the change in pH below bryophytes with the pH below neutral cover from the first control from the experimental conditions by a Wilcoxon rank-sum test.

## 5. Conclusions

Bark pH is one of the ecological factors that affects the occurrence of bryophytes. Our results support the idea that bryophytes can contrarily change the pH of the bark. The pH of the bark results from many forces, including long-lasting moisture and settling of dust leading to alkalization, washing with flowing water, and acid rains leading to acidification. These forces lead to low pH in low inclination trunks and high pH in high inclination of trunks. Epiphytic bryophytes may bring balance to those forces by decreasing the velocity of flowing water and increasing dust settling, further alkalizing trunks by their very presence and acidifying them by their biological reactions. Thus, they may influence the presence of other epiphytic biota by inclination-dependent interactions. 

## Data Availability

All authors agree with MDPI Research Data Policies.
